# Symptom interpretation and health care seeking in ovarian cancer

**DOI:** 10.1186/1472-6874-11-31

**Published:** 2011-06-23

**Authors:** Lene Seibaek, Lone K Petersen, Jan Blaakaer, Lise Hounsgaard

**Affiliations:** 1 Department of Obstetrics and Gynaecology, Aarhus University Hospital, Skejby, Aarhus, Denmark; 2 Research Unit of Nursing, Faculty of Health Sciences, University of Southern Denmark, Odense, Denmark

## Abstract

**Background:**

Ovarian cancer is the leading cause of death among women suffering from gynaecological malignancies in the Western world. Worldwide, approximately 200,000 women are diagnosed with the disease each year. This article deals with the health care seeking and symptom interpretation process among Danish women, who have a very high mortality rate.

**Methods:**

The health seeking and symptom interpretation process was analysed via combining study methods. The material consisted of registry data dealing with the use of public health care and hospital services of Danish women, newly diagnosed with ovarian cancer. These results were combined with findings from semi-structured qualitative research interviews on women's bodily experiences with symptom development.

**Results:**

A number of 663 Danish women with ovarian cancer attended 27 different kinds of primary health care providers in a total of 14,009 visits during 2007. The women also had 6,214 contacts with various hospitals, and obtained 562 different diagnoses.

From the main theme "Women's experiences with the onset of symptoms" three sub-themes were identified: "Bodily sensations", "From bodily sensation to symptom", and "Health seeking and treatment start". In all cases the General Practitioner represented the first contact to public health care, acting as gate-keeper to specialist and hospital referral.

The women were major users of public health care throughout the diagnostic process and subsequent treatment. All women held personal knowledge concerning the onset of their symptoms. The early symptoms of ovarian cancer might be uncharacteristic and non-disease-specific when interpreted as personal experiences, but they had similarities when analysed together.

**Conclusions:**

Diagnostic delay in ovarian cancer seems far from being exclusively a medical problem, as the delay proved to be influenced by organisational, cultural, and social factors, too. Initiatives facilitating the diagnostic process and research concerning the selection of individuals for further investigation are indicated. The way in which the women interpreted their symptoms was influenced by their personal experiences, their cultural, and their social background. This became crucial to the diagnostic process. These issues need to be explored through further research on women's experiences during the diagnostic process.

## Background

This paper reports a study aiming to analyse the health seeking process and the individual symptom interpretation process in women with ovarian cancer.

Each year more than 200,000 women are diagnosed worldwide with ovarian cancer; which is the leading cause of death among women suffering from gynaecological malignancies in the Western world [1]. The early FIGO (International Federation of Gynaecology and Obstetrics) stages I-II of ovarian cancer have a very good prognosis with a five-year survival of 80-90%, but the five-year survival declines substantially to about 25% in the advanced FIGO stages III-IV. Early detection is therefore of crucial importance so that treatment can be started when the disease is still in its early stages [2].

The international community of medical professionals has been addressing this issue for decades, unfortunately without achieving an adequate solution to the problem [3]. First and foremost, the non-disease specificity of the symptoms has made ovarian cancer very difficult to detect in time and, although clinical trials are ongoing, valid screening programmes are not yet available [4]. Ovarian cancer has thus been perceived as "a silent killer"; an assumption which is still leading to misdiagnosis and delay in numerous cases. However, previous studies have identified a symptom cluster preceding diagnosis, and information campaigns as well as self-monitoring programmes for women at risk are being developed and tested [5-8]. Studies by Goff, Bankhead, and Hamilton have identified symptoms presented in general practice which are independently associated with ovarian cancer, of which the most significant are abdominal distension, increased urinary frequency, and abdominal pain [9-11]. Against this background it should be evident that the disease, no matter how hard it is to obtain an early diagnosis, can no longer be described "a silent killer". On the other hand, it is persistently, and with some justification, argued that referring all women with the above mentioned symptoms to specialist care would put a severe strain on public health care, and lead to many needless worries among healthy women.

With 16.9 cases per 100,000 and a mortality-incidence ratio on 74.4% Danish women have one of the world's highest incidence and mortality rates for ovarian cancer [12]. Considerable efforts have therefore been made to improve the treatment, and in 2007 the government issued a guarantee of free and fast treatment of all cancers [13-17]. Since then Danish women have a maximum wait of two weeks from referral to surgery, and treatment has been centralised, specialised, and standardised into fast-track programmes [18]. However, even though treatment options have improved the five-year survival rate has continued to be unsatisfactorily low. This is most likely because more than half of the women are still being diagnosed in advanced stages [1,2,19]. In 2007 the stage distribution was 25% stage I, 7% stage II, 48% stage III, 18% stage IV, and 2% having an unspecified stage. The diagnostic distribution was 72% ovarian cancer, 25% borderline, and 3% cancer of the fallopian tubes [20,21].

Steps to optimise and speed up the diagnostic procedures have been initiated: the imaging procedures in primary care have been improved, and the concept of patient delay in cancer diagnostics has been subject to intensified investigation [22]. A recent work by Andersen et al. suggests that the symptom interpretation process for cancer patients does not solely depend on the specific disease or the presence of specific symptoms. It also depends on the way in which individuals understand their bodily sensations, on the organisation of health care, and on health politics [23].

### Ethics and consent

The study was approved by the Danish Data Protection Agency (file no. 2007-41-1640). In keeping with the rules of the Helsinki Declaration on voluntariness and anonymity the women received both verbal and written information on the project, before they gave their written consent [24]. In accordance with the Central Denmark Region's Committees on Biomedical Research Ethics the study needed no further approval.

## Methods

The material consisted of registry data from Danish women newly diagnosed with ovarian cancer, concerning their use of public health care and hospital services during the year of diagnosis (2007). These results were subsequently combined with findings from semi-structured qualitative research interviews, which were conducted with women undergoing ovarian cancer surgery in late 2008 and early 2009, dealing with their personal experiences with symptom development [25].

### Registry data and statistics

The population consisted of women with borderline ovarian tumours, ovarian cancer, and cancer of the fallopian tubes, who were registered in the Danish Gynaecological Cancer Database (DGCD) in 2007 [26,27]. It was possible to combine data from various registers as all Danish citizens and permanent residents have a unique civil registration number (CRN) [28].

Data on the use of health care services in primary health care originated from the National Health Insurance Service Registry. A registration by CRN in this register indicates a contact with a registered healthcare provider. Every contact included a number of visits, which related to this specific contact. The database provided information on the number of visits, as they were linked to an electronic reimbursement system. The care provider was unfortunately not obliged to state neither the reason for nor the date of the visits in the database.

Data on the use of hospital services originated from the National Patient Registry where data on all inpatient, outpatient, and emergency contacts with Danish hospitals were collected.

Data were summarised, displayed, and reported in frequencies, percentages, means, medians, and standard deviations (SD).

### Interview data and analyses

The participants were women who underwent surgery for borderline ovarian tumours and ovarian cancer at a Danish national centre for surgical treatment of gynaecological malignancies during 2008-2009. They were selected strategically to represent the population of women newly diagnosed with ovarian cancer with regard to age in years, final diagnosis, and stage, cohabitation, socio economic group, number of children, and housing (Table 1)[12,29].

**Table 1:** Interviewee characteristics

#	Age	Diagnosis	Stage	Cohabitation	Socioeconomic group	Children	Housing
1	51	Ovarian cancer	IIIC	Single	Employee	0	Town house

2	29	Ovarian cancer	IA	Cohabiting	Student	0	Apartment

3	62	Ovarian cancer	IV	Married	Retired	2	Apartment

4	79	Ovarian cancer	IA	Widowed	Retired	1	Apartment

5	57	Borderline tumour	-	Single	Retired	1	Other (cottage)

6	66	Ovarian cancer	IIIC	Married	Retired	2	Single family house

7	61	Ovarian cancer	IIIC	Married	Civil servant	3	Official residence

8	72	Ovarian cancer	IIIC	Widowed	Retired	2	Apartment

9	60	Ovarian cancer	IC	Married	Employee	2	Single

10	51	Ovarian cancer	IV	Married	Employee	3	Single

Each woman was interviewed twice. The first interview took place at the hospital ward the evening before surgery. The second interview took place eight weeks later in the interviewee's private home. In this way a balanced interview setting was obtained. The interviews followed a semi-structured interview guide (Table 2).

**Table 2:** Semi-structured interview guide

Questions during the preoperative interview
How have you been feeling since surgery was decided on?
What have you been doing these past days?
What are your thoughts about undergoing surgery?
What are your thoughts about the time after the operation?

**Questions during the postoperative interview**

How do you feel at the moment?
How did you experience your discharge?
In which way has the disease and treatment impacted your life?
What are your thoughts about the future?

All the interviews were conducted by the first author, who had a professional background as a specialist nurse within the field of gynaecological cancer, but was not a care provider during the study period. The interviews were digitally recorded, transcribed verbatim, and analysed. The qualitative research software NVivo8 was used to systematise the findings, and prepare the text for analysis.

By using a phenomenological-hermeneutic text interpretation methodology, the interview findings were systematically identified and put into meaning structures. Subsequently they were interpreted and discussed [30]. The text interpretation took place on three analytic levels: the naïve reading, the structural analysis, and the critical analysis and discussion. Through the intentionally naïve reading and rereading of the text an initial overview of the text as a whole and an interconnected understanding of the meaning embedded in the text was created. This was followed by structural analysis and further operationalisation of the findings, in a dialectic process which moved between quotations "what the text said" and meaning condensation "what the text spoke about". Pursuant to the conception of phenomenological hermeneutics as an argumentative discipline the findings were subsequently validated through interpretation and discussion.

## Results

### Registry data

A population of 666 women constituted the data material.

### The use of health care services

The women (n:663) had a total of 2,953 primary health care contacts with 27 different types of registered primary health care providers within the year of diagnosis. Three women had no contact with primary health care, neither before, nor after their surgery. Every contact contained a number of visits, which were related to the specific contact. The median number of visits contained in each contact was 4.8 (range 1-62). A total of 14,009 visits were registered in the study population (mean: 21).

The ten most frequently used health care providers were listed in Table 3. The most frequently used primary health care provider was by far the General Practitioner (GP) who was involved in 69.1% of the contacts. This was followed by dentists, who had 14.3% of the contacts. Gynaecologists were involved in 5.6% of the contacts, whereas psychologists were involved in less than one % of the primary health care contacts.

**Table 3:** The most frequently used health care providers

Health care provider	Number	(%)	Mean visits per contact	SD of visits
General Practitioner	1 964	(69.1)	5.6	5.7

Dentist	407	(14.3)	1.7	0.9

Gynaecologist	159	(5.6)	2.2	1.5

Ophthalmologist	120	(4.2)	1.9	1.6

Physiotherapist	64	(2.3)	9.4	11.6

Dermatologist	38	(1.3)	3.1	2.3

Chiropractor	37	(1.3)	6.3	5.0

Practising surgeon	22	(0.8)	1.6	0.7

Psychologist	17	(0.6)	6.4	5.9

Rheumatologist	14	(0.5)	2.4	1.6

Table 4 provides a detailed presentation of the contacts with the GP, demonstrating that 82% of the consultations were daytime consultations, and that 53% were telephone consultations.

**Table 4:** Types of contacts and visits to the General Practitioner

Contact to GP	Number	(%)	Mean visits per contact	SD of visits
Consultation (Day)	642	(41)	7.4	5.2

Consultation (Evening)	97	(6)	1.2	0.7

Telephone consultation (D)	621	(41)	7.8	6.8

Telephone consultation (E)	186	(12)	1.9	1.9

### Referral to hospital services

The sample of 666 women was registered as users of hospital services with a total of 6,214 contacts. Of these 44% were inpatient contacts (n: 2,689), 54% were outpatient contacts (n: 3,378), and 2% were emergency ward contacts (n: 147).

A total of 562 different initial or final diagnoses were used. According to the Danish version of the International Classification of Diseases ICD-10 [31], the referral diagnoses related to potential ovarian cancer were: DZ031 "observation due to suspicion of malignant tumour," DZ031K "observation due to suspicion of malignant tumour in female genitals," and DZ038 "observation due to suspicion of other diseases or conditions."

Twenty-one% (n: 140) of the women were referred by DZ031K which is the most specific ovarian cancer diagnosis code; 39% (n: 259) were referred by DZ031 and three % (n: 21) were referred by DZ038. The remaining 37% (n: 246) had various other referral diagnoses.

### Interviews

**Figure 1:**
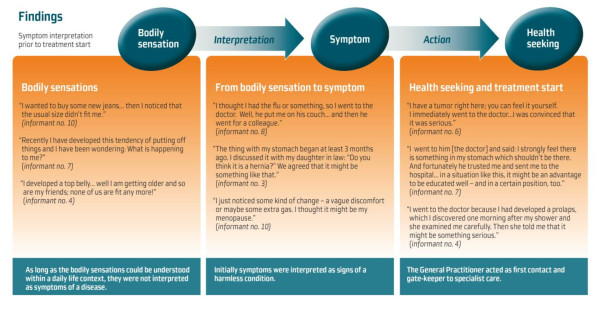
**Symptom interpretation prior to treatment start**.

A total of nineteen qualitative research interviews were conducted preoperatively (n: 10) and postoperatively (n: 9) with ten women aged 29-79. Each interview lasted from 19 to 111 minutes (median: 43 minutes). All of the invited women accepted to participate in the study. However, one woman was interviewed only preoperatively due to her death shortly after the surgical procedure. The interviewees were in the following referred to as informant 1-10 (Figure 1).

### Interview analysis

The intentionally naïve reading of the text creating an overview of the text as a whole and an interconnected understanding of the content, showed that although individually expressed all women had experienced symptoms, which were independently associated with ovarian cancer such as abdominal distension, increased urinary frequency and abdominal pain, before health seeking. This main finding constituted the theme "Women's experiences with the onset of symptoms." This theme primarily related to the women's personal symptom interpretation prior to diagnosis and treatment start.

The further analysis and operationalisation of this theme constituted three sub-themes which related to various steps in the individual symptom interpretation process: "Bodily sensations", "From bodily sensation to symptom", and "Health seeking and treatment start". These sub-themes were identified through patterns embedded in the text, consisting of the women's personal actions and experiences. The sub-themes are presented in Figure 1, where a flow chart illustrates that the bodily sensations had to be interpreted as symptoms, in order to bring about initial health care seeking and subsequent specialist or hospital referral.

The first sub-theme "Bodily Sensations" dealt with the every-day-life situations, in which the women had first noticed ongoing changes in their bodies; this was however still without realising that these could be signs of any disease. As illustrated by informants 10, 7, and 4 these initial changes in bodily perception were recognised for instance when buying new clothes, as a sign of old age when being together with peers, or simply during the conduct of their daily life.

The second sub-theme "From bodily sensation to symptom" dealt with women's actions and personal reflections, when the persistent bodily sensations were interpreted as symptoms. As illustrated by informants 8, 3, and 10, the symptom presentation was not always related to a gynaecological condition: Informant 8 thought she had the flu, and Informant 3 thought she had a hernia. As Informant 10 interpreted her symptoms originating from her menopause, and as she did not want any medication for this condition, she refrained from health care seeking for several months.

The third sub-theme "Health seeking and treatment start" dealt with the informants' presentation of symptoms and their interaction with health care professionals. The sub-theme described the impact of educational and social status on the interaction, as the women with high social status (i.e. Informant 7) were more likely to speed up health seeking and ask their GP's for specialist or hospital referral (Figure 1).

## Discussion

### Health care seeking and referral to hospital services

In Denmark the GP is the predominant provider of care for non-serious ailments. Consequently the GP is acting as gatekeeper to specialist services, including referral to a gynaecologist [19].

The Danish adult population attended primary health care providers on average 11.6 times in 2007. Correlation between gender and employment showed that males had an average of nine visits, and females an average of 14 visits that year. The most frequent visits were made by female pensioners [32]. Having a mean of 21 visits the newly diagnosed women with ovarian cancer had a very high frequency of contacts with the GP. Still they were seldom referred to a specialist. Furthermore 53% of the consultations took place via the telephone, which excluded physical examination or simply observation (Table 4). Under the existing registration process information on the exact time, and the exact cause of each visit was not available. It was a weakness in the registry data, and the extent to which these visits had any relation to the ovarian cancer diagnosis remained unknown.

It is striking that more than five visits to the GP were required prior to the GP becoming suspicious of an ovarian malignancy, and that only 24% of the women had visited a gynaecologist before they were diagnosed. Furthermore, only 21% were referred to a gynaecological cancer centre due to suspected malignancy of the female genitals [14,15]. This is a very low referral rate, as the UK referral rate is on 31% even though the UK represents another prosperous Western country, struggling with inadequate results in ovarian cancer survival [33]. Goff et al. found that gynaecologists, physicians involved in teaching, and GP's in group practices were significantly more likely to provide referral and testing in relation to an ovarian cancer diagnosis [34]. These results highlight the need to facilitate the diagnostic procedures, and to develop clinical pathways in relation to ovarian cancer diagnostics.

At present, the difficulties in diagnosing ovarian cancer place a severe burden on the lives of the women as well as on the health care system, in terms of high costs and poor results. It cannot be denied that introducing a quick specialist referral procedure might temporarily intensify this strain, in terms of an increased use of specialised health care resources. However diagnosing and treating the disease in less advanced stages would substantially improve the chances of survival as well as improve the women's quality of life. Against this background we agree that research in primary care concerning the selection of patients for further investigation is urgent, as suggested by Hamilton et al. [11,35].

### Symptom development: the woman's perspective

The interview findings were analysed as recalled experiences with the development of symptoms, therefore recall bias could not be eliminated. Furthermore, the lack of a valid measure of patient delay makes the pre-diagnostic period particularly difficult to investigate [22,23]. On the other hand, the pattern in findings of the present study could be independently confirmed in other studies [11,36]. As long as the bodily sensations could be explained within a daily life context, they were not interpreted as symptoms of any disease at all. This was illustrated by Informant 10, who did not notice her growing stomach until it affected the size of her clothing. Informant 3 and her family misinterpreted the rather visibly distended abdomen as a symptom of a harmless condition, as they all agreed that it was probably a hernia. Both cases resulted in an ovarian cancer stage IV diagnosis.

However, the diagnosis-seeking processes did not exclusively involve personal experiences. How and when the women interpreted their bodily sensations as symptoms of a specific disease was also influenced by their cultural and social backgrounds [37]. When the bodily sensations were actually identified as symptoms, they were initially interpreted as symptoms of some kind of harmless condition, such as the menopause. Consequently it was not until late in the process the women realised that the symptoms were signs of a potentially serious disease. This was clearly demonstrated by Informant 6, who could suddenly feel the tumour directly below her skin. In all cases the GPs represented the women's first contact with public health care and acted as the gate-keepers to specialist and hospital referral.

The findings suggest that the way the women presented their symptoms was crucial to the course of their diagnostic process. Through the interviews at least three different diagnostic scenarios were identified: In the first scenario the woman and the GP both misinterpreted the symptoms causing diagnostic delay (Informant 8). In the second scenario the GP referred the woman to immediate specialist investigation after having performed a physical examination (Informant 4), and in the third scenario the GP and the woman both were aware of a potentially serious condition, but further investigation was nevertheless delayed (Informant 7). The preferred situation was clearly represented by Informant 4, who was diagnosed in a localised stage and treated with surgery alone.

### The impact of social status

Studies by Hannibal et al. and Hansen et al. have demonstrated that in ovarian cancer as well as in many other diseases, the living conditions have a significant impact on survival. The systematic distortion in the one-year survival of women with ovarian cancer, with respect to their level of education, disposable income, early retirement, marital status, and co-morbidity, clearly indicated inequality in the access to treatment even though it was free [12,38]. Several studies have demonstrated that individuals having a high socio-economic status are far more capable of seeking and sustaining relevant treatment [39,40]. This finding was confirmed via the interviews. Informant 7 highlighted: "And fortunately he trusted me and sent me to the hospital... in a situation like this it might be an advantage to be educated well - and in a certain position, too".

## Conclusions

In ovarian cancer diagnostic delay appears to be far from exclusively a medical problem. The delay is also influenced by organisational, cultural, and social factors.

In the current study the women paid many visits to GP prior to specialist and hospital referral for further investigation. Women of higher socio-economic status appeared to be more capable of seeking and sustaining treatment. In a Danish context the referral procedure from GP to cancer specialist requires a referral diagnosis. Such a diagnosis can be difficult to provide, since the presentation of symptoms is vague and non-disease-specific. Initiatives facilitating the diagnostic procedures and improving the diagnostic pathways are therefore strongly needed; preferably in addition to research concerning the selection of individuals for further investigation. Finally GPs should be given the alternative possibility of referring patients with vague and non-disease-specific symptoms, to clinics specialised in diagnosing cancer per se.

All the interviewed women held personal knowledge of their symptom development. The way in which they understood and presented their symptoms was influenced by their personal experiences, and their cultural and social background. Both symptom interpretation and symptom presentation were crucial to the outcome of their diagnostic process. The early symptoms of ovarian cancer which appeared to be non-disease-specific when interpreted as individual experiences, showed strong similarities when analysed together. We therefore suggest further research on women's personal experiences of symptom development.

## Competing interests

The authors declare that they have no competing interests.

## Authors' contributions

LS collected and analysed the statistical data, conducted and transcribed the interviews, interpreted the findings, and initiated the preparation of this manuscript. LKP, JB and LH contributed to the design of the study, the analyses and interpretation of the data, and revised the manuscript. All authors read and approved the final manuscript.

## Pre-publication history

The pre-publication history for this paper can be accessed here:

http://www.biomedcentral.com/1472-6874/11/31/prepub
